# Primary Renal Synovial Sarcoma

**DOI:** 10.1155/2011/810184

**Published:** 2011-08-11

**Authors:** Mehmet Gulum, Ercan Yeni, Murat Savas, Ilyas Ozardali, Ismail Ozdemir, Dilek Mil, Adem Altunkol, Halil Ciftci

**Affiliations:** ^1^Department of Urology, School of Medicine, Harran University, 63100 Sanliurfa, Turkey; ^2^Department of Pathology, School of Medicine, Harran University, 63100 Sanliurfa, Turkey; ^3^Department of Urology, Sanliurfa Education and Research Hospital, 63200 Sanliurfa, Turkey; ^4^Department of Pathology, Sanliurfa Education and Research Hospital, 63200 Sanliurfa, Turkey

## Abstract

Synovial sarcomas are generally deep-seated tumors that most often occur in the proximity of large joints of adolescents and young adults. We describe two cases of primary renal synovial sarcoma that were treated successfully by radical nephrectomy. Synovial sarcoma originating from the kidney is extremely rare and the histogenesis is uncertain. Surgical resection and ifosfamide based chemotherapy are the mainstay for the management of renal synovial sarcoma. Fewer than 40 patients have been described in the English literature. Physicians should be aware of the possibility of malignancy in cystic renal masses and raise the suspicion of synovial sarcoma, especially when patients with renal masses are a young adult.

## 1. Introduction

Synovial sarcomas (SSs) are generally deep-seated tumors that most often occur in the proximity of large joints of adolescents and young adults. They account for about 5% to 10% of adult soft-tissue sarcomas [1]. They can be observed in unexpected sites, such as thoracic and abdominal wall [2], head and neck region, including pharynx and larynx [3], retroperitoneum [4], and bone [5] as well as visceral organs, such as lung [6], pleura [7], or prostate [8]. Primary renal SS is a rare tumor first described by Argani et al. in 2000 [9]. Since then, cases have been sporadically reported [10, 11]. To date, knowledge about this rare malignancy remains limited. Its presentation is similar to that of other renal tumors. The diagnosis is confirmed by immunohistochemical stain or cytogenetic study [11]. 

We describe two cases of primary renal synovial sarcoma that were treated successfully by radical nephrectomy.

## 2. Case  1

**Figure 1:**
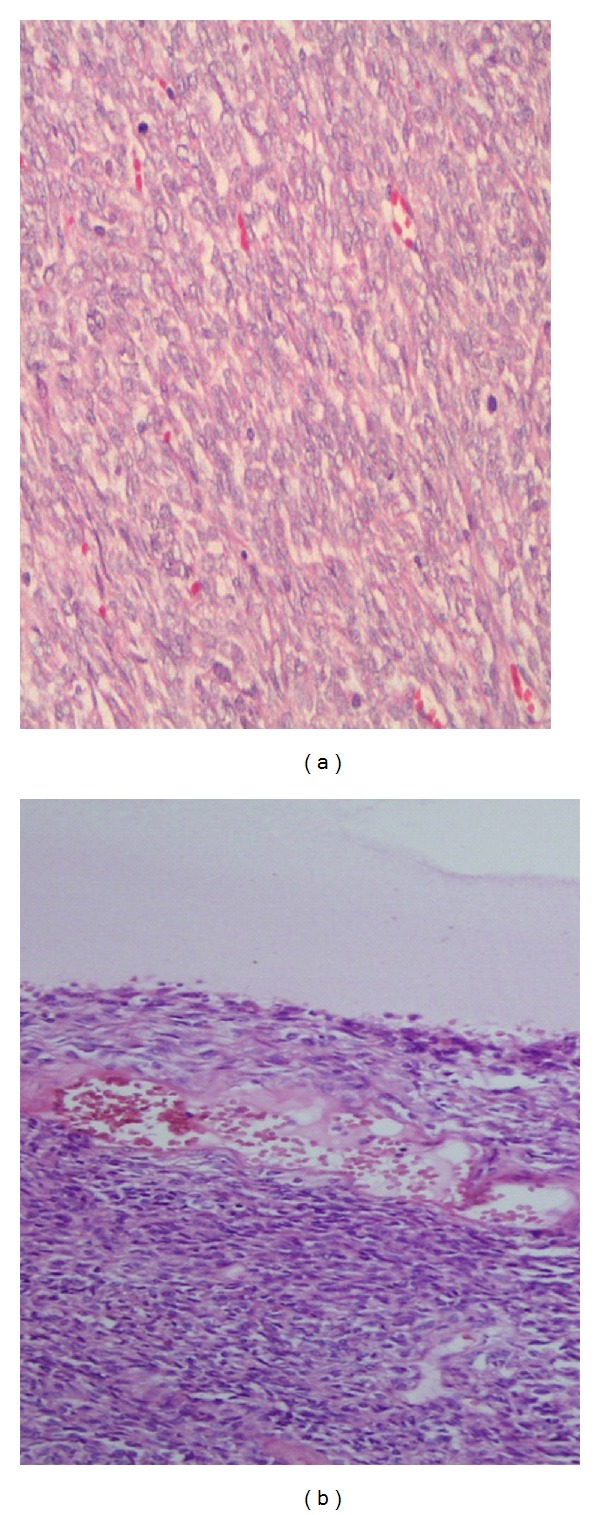
Histologic examination of tumoral tissues composed of solid cellular conglomerates of monomorphic spindle cells with nonuniformly bounded cytoplasm in large areas and fascicles with cystic structures settled among them.

A 18-year-old female presented with about a 1-month history of right flank pain. A 7 cm mass revealed by ultrasonography in the mid-pole of left kidney. In the left renal mid-pole, 7 × 7 × 6 cm low-density mass was reported on computed tomography images. Left TCC was suspected. The patient subsequently underwent left nephroureterectomy. Grossly, in intraoperative observation, the tumor was approximately 7 cm, originating from left kidney mid-pole. During surgery, the gross invasion of surrounding tissues and regional lymphadenopathy was not noted. Pathologic confirmation was performed by immunohistochemical methods. In histologic examination, solid cellular islets was observed on cross-sections of the tumoral tissue stained with hematoxylin-eosin (Figure 1(a)). No extracapsular extension was reported. Chemotherapy was recommended, but it was not accepted by the patient. No evidence of recurrence was found at the 15-month followup. Immunohistochemical findings are listed in Table 1.

## 3. Case  2

**Figure 2:**
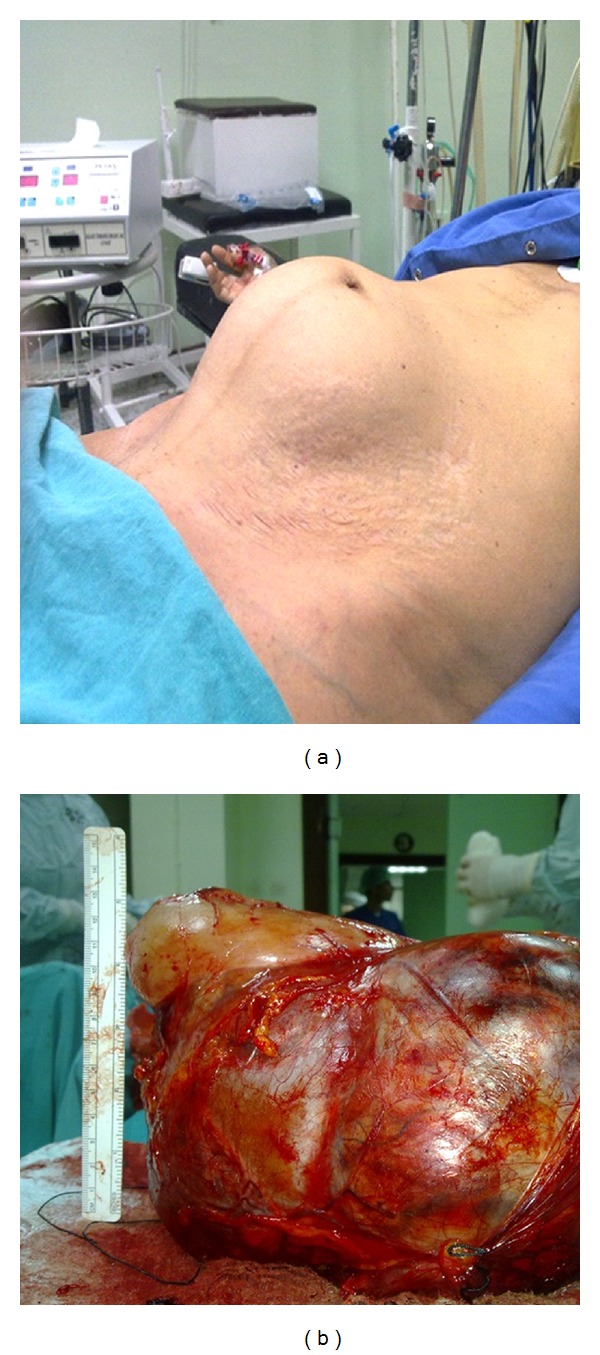
Macroscopic views of the case two.

A 68-year-old woman presented with a 3-month history of abdominal distension (Figure 2(a)) and right flank pain. Magnetic resonance imaging revealed a heterogeneous enhancing soft-tissue mass originating from the upper pole of the right kidney which exhibited a distinct press on vena cava inferior, repelled renovascular structures, and aorta. In T2A images, the mass consisted of cystic necrotic structures in a patchy manner and had a capsule. No extracapsular extension was reported.

The patient subsequently underwent right nephroureterectomy. Grossly, in intraoperative observation the tumor was approximately 20 cm originating from right kidney upper pole and stuck on vena cava inferior. The resected tumor appeared irregular in shape and measured 25 × 15 × 7 cm (Figure 2(b)). It showed an infiltrative growth pattern to surrounding renal tissue. Cut surface of the mass was grayish white coloured and had with focal hemorrhage and yellow-gray necrosis. Histologic examination of tumoral tissue composed of solid cellular conglomerates of monomorphic spindle cells with nonuniformly bounded cytoplasm in large areas and fascicles with cystic structures settled among them (Figure 1).

The patient underwent 3 cures of IMA (ifosfamide (I), mesna (M), and doxorubicin (A)) chemotherapy each of which applied in three days. Doxorubicin 60 mg/m^2^ (70 mg) only the first day, ifosfamide 2500 mg/m^2^ (3000 mg) 1–3 days, and mesna 2500 mg/m^2^ (3000 mg) 1–3 days. The patient had no evidence of recurrence at the 11-month followup. Immunohistochemical findings are listed in Table 1.

## 4. Discussion

Synovial sarcoma is the fourth most common soft-tissue sarcoma, which primarily develops in the limbs of young people. However, primary renal sarcomas are rare [1–3]. Synovial sarcoma originating from the kidney is extremely rare, and the histogenesis is uncertain. Fewer than 40 patients have been described in the English literature [1–11]. It affects young individuals of both genders. Cases are between ages 17–72. The average age was 38.5 years, and male predominance was noted with a male-to-female ratio of 1.7 : 1.4 by Chen et al. in a review of 19 case reports [12]. Histologically, primary renal synovial sarcomas consist of plump spindle cells with minimal cytoplasm, active mitotic figures, and tubular cells. Cysts are commonly present and are lined with epithelial cells that possess eosinophilic cytoplasm with apical nuclei that create a hobnail appearance [11, 13]. In general, there are no clinical or imaging features that can contribution definitive preoperative diagnosis. The diagnosis always requires pathologic confirmation. Synovial sarcomas generally stain positively for cytokeratin, vimentin, bcl-2, and epithelial membrane antigen.

The prognosis of primary renal synovial sarcoma is obscure due to the limited number of reported cases. From previously published data, renal synovial sarcomas are believed to have aggressive clinical courses and poor outcomes [14, 15]. Surgical resection and ifosfamide-based chemotherapy are the mainstay for the management of renal synovial sarcoma [16]. One case reported by Schaal et al. showed reply to a regimen using ifosfamide and adriamycin [17].

 In conclusion, renal synovial sarcoma is rare and commonly affects young adults. Although cystic renal mass was showed only in the one of our two cases, it was also reported frequently for renal synovial sarcoma in the literature. Physicians should be aware of the possibility of malignancy in cystic renal masses and raise the suspicion of synovial sarcoma, especially when patients with renal masses are a young adult.

## Figures and Tables

**Table 1:** Clinical and immunohistochemical data of the two cases.

	Age (yr)/Sex	Tumor size (cm)	Component	Bcl-2	CK	EMA	Vimentin	CD99
Case 1	18/F	7 × 7 × 6	Epithelial	Focal (+)	+	+	+	Focal (+)
			Spindle	−	−	−	+	−

Case 2	68/F	25 × 15 × 7	Epithelial	Focal (+)	+	+	+	Focal (+)
			Spindle	−	−	Focal (+)	+	−

CK = cytokeratin (monoclonal, 1 : 50; Dako); EMA = epithelial membrane antigen (monoclonal, 1 : 200; Dako); Vimentin = vimentin (monoclonal, 1 : 200; Dako); CD99 = CD99 (monoclonal, 1 : 50; Dako).
